# The Roles of Macrophages in Heart Regeneration and Repair After Injury

**DOI:** 10.3389/fcvm.2021.744615

**Published:** 2021-10-25

**Authors:** Ying Gao, Ningjing Qian, Jingmiao Xu, Yaping Wang

**Affiliations:** ^1^Department of Cardiology, The Second Affiliated Hospital, Zhejiang University School of Medicine, Hangzhou, China; ^2^Cardiovascular Key Lab of Zhejiang Province, Hangzhou, China

**Keywords:** inflammation, macrophages, subsets, heart injury, heart repair

## Abstract

Although great advances have been made, the problem of irreversible myocardium loss due to the limited regeneration capacity of cardiomyocytes has not been fully solved. The morbidity and mortality of heart disease still remain high. There are many therapeutic strategies for treating heart disease, while low efficacy and high cost remain challenging. Abundant evidence has shown that both acute and chronic inflammations play a crucial role in heart regeneration and repair following injury. Macrophages, a primary component of inflammation, have attracted much attention in cardiac research in recent decades. The detailed mechanisms of the roles of macrophages in heart regeneration and repair are not completely understood, in part because of their complex subsets, various functions, and intercellular communications. The purpose of this review is to summarize the progress made in the understanding of macrophages, including recent reports on macrophage differentiation, polarization and function, and involvement in heart regeneration and repair. Also, we discuss progress in treatments, which may suggest directions for future research.

## Introduction

Heart disease is one of the leading causes of death worldwide. Previous studies have predicted that there will be over 8 million adults suffering from heart failure within about 10 years (1). Heart disease is also a great financial burden to society. According to the recent data on heart disease and stroke, the costs of cardiovascular disease and stroke were $351.3 billion from 2014 to 2015 in the United States (2). While in China, the total cost of hospitalization for ischemic heart disease was ¥111.982 billion in 2018, with an average annual growth rate of over 20% (3). Diseases like arrhythmia, valvulopathies, and cardiomyopathies can lead to heart failure and ultimately the loss of mobility. Due to minimal regenerative capacity, mature cardiomyocytes have little ability to compensate for the loss of cells following ischemia, mechanical stress, hypothermia, and other injuries. Therefore, to maintain normal pumping function and integrity under such circumstances, cardiomyocytes tend to become thicker and longer with increased contractility. Furthermore, cardiac remodeling is also characterized by an abnormal increase in the numbers of cardiac fibroblasts and extracellular matrix (ECM) (4, 5). However, when myocytes are overloaded, the heart will ultimately exhibit contractile and diastolic dysfunction. At present, there are many ways to treat heart disease, including drugs, surgery, and heart transplantation, whereas each of these has limitations in terms of therapeutic effectiveness and economic cost. Therefore, the identification of new therapeutic targets seems to be crucial for slowing disease progression and improving the quality of life. The roles of inflammation and macrophages have received much attention. This review explores the current progress in understanding the relationship between macrophages and myocardial regeneration, and summarizes the potential therapeutic targets for stimulating the heart muscle repair following cardiac injury.

## Heart Regeneration and Repair

Cardiomyocytes were used to be identified as non-renewable cells with low proliferative ability. It seems to be difficult for cardiomyocytes to repair the injured heart by regeneration. However, in 2002, Poss et al. first reported that 1- to 2-year-old adult zebrafish could completely regenerate their hearts over a 2-month period instead of producing scar tissue in response to apical resection (6, 7). In addition to fish, another experimental model was reported by Porrello et al. (8). The same surgery was performed on 1-day-old mice and complete heart regeneration was observed at 21 days after the operation (8). In other words, mammals could also have the capacity to replenish cardiomyocytes and regenerate myocardium. However, this regenerative ability was extremely attenuated 7 days after birth (7, 8). In humans, mitosis has been observed in cardiomyocytes of adult hearts under both normal and pathological conditions, thus indicating myocyte proliferation and the potential of myocardial renewal (9, 10). The proportion of cardiomyocytes undergoing mitosis was reported to be 0.3–1%, and it could be slightly higher in the hearts after myocardial infarction (11). Considering the lower rate of regeneration in the adult mammalian heart, fibrosis and hypertrophy still seem to be the dominant processes during the cardiac repair.

## The Relationships Between Inflammation, Heart Regeneration, and Repair

Inflammation, defined as the defensive reactions to external or internal injury, is a process in which inflammatory cells infiltrate the injured tissue, remove damaged cellular debris, release various chemokines and cytokines, and stimulate cells like fibroblasts to participate in tissue repair. In the heart, inflammation plays a central role in post-injury repair (12). When an ischemic injury occurs, acute inflammation is initiated within a few hours (13–15). Leukocytes, mainly neutrophils, then accumulate in the damaged area, remove cell fragments, secrete cytokines to modify the microenvironment, and recruit other immune cells (16, 17). In previous studies, early and acute inflammation was thought to play a negative role in myocardial repair, which resulted in collagen deposition and scar formation, and even a decrease in contractility. However, in recent decades, it has gradually become apparent that treatments that suppress the immune system, such as dexamethasone (18) or microinjection of immunogenic particles (19), may damage the mouse heart following cardiac ischemic injury. If neonatal mice received such therapy, the regenerative capacity of the heart would undoubtedly be greatly reduced. Consistent with this concern, suppression of interleukin-6 (IL-6) and its downstream target, signal transducer and activator of transcription 3 (STA3), resulted in greatly reduced cardiomyocyte proliferation following apical resection of hearts in 1-day old mice (18). Therefore, acute inflammation can be considered a double-edged sword; it is necessary to initiate proliferation and regeneration, but an excessive immune response can amplify scar tissue formation and lead to irreversible damage and dysfunction. Chronic inflammation has been shown to play a vital role in cardiac remodeling and dysfunction following heart failure, but therapies that suppress the inflammatory response have had a little curative effect (20, 21).

## Macrophages and Heart Regeneration and Repair

### Origins, Development, and Differentiation of Macrophages

Macrophages are a part of the innate immune system and mononuclear phagocytic population, participating in phagocytosis, chemotaxis, secretion, and antigen presentation for immune defense and tissue healing (17, 22–24). With the development of tools like lineage tracing and fate mapping, researchers began to realize that contrary to the idea that macrophages are derived from blood monocytes, different macrophage lineages were resident in tissues, including precursors of monocytes in the yolk sac and fetal liver (25–28). It is known that the generation of macrophages in rodents can generally be described as occurring in three stages, including primitive hematopoiesis in the yolk sac, followed by differentiation into erythro-myeloid progenitors (EMPs) and hematopoietic stem cells (HSCs) (29). Macrophages from both the yolk sac and HSC can be found in the heart, and their proportions are stable in the healthy tissue (29, 30).

To highlight the different roles in the inflammatory response of these subsets of macrophages, Mills has in recent decades developed the concept of M1 and M2 macrophages in mice, where M1 acts to inhibit and M2 acts to heal (31, 32). Later, based on this framework, researchers began to combine the M1 type with macrophages that are classically activated by interferon γ (IFN-γ) and lipopolysaccharide (LPS) *in vitro* and combine the M2 type with those that are alternatively activated by cytokines like interleukin-4 (IL-4) and interleukin-13 (IL-13) *in vitro* (33–38). On the one hand, this classification is not entirely consistent with the ideas of Mills who defined macrophages based on their function, and the macrophage types defined in these two ways actually have different gene signatures as shown by comparing transcriptomes between the M1/M2 and classically/alternatively activated types (39, 40). On the other hand, it is an oversimplification to describe the spectrum of macrophage subsets using only two categories (34, 41–43), which neglect the diversity of macrophages in distinct tissues and conditions (33, 44, 45). Some suggest that M1 and M2 are more like two extremes of macrophage polarization and that the observation of the complete process is limited by experimental methods (46). Nevertheless, this classification provides a basic framework for further studies of the dynamic differentiation of the macrophages.

In contrast to blood cells, macrophages can also be categorized as resident and monocyte-derived types. This kind of classification emphasizes their different origins and functions. In general, almost all resident macrophages originate from yolk sac progenitor cells that have the ability to self-renew until adulthood, although their numbers and proliferative ability decrease with age (30). Tissue-resident macrophages play a crucial role in embryogenesis, participating in the formation of various structures (29). Monocyte-derived macrophages, which usually originate from HSCs, can be replaced by adult cells that are also monocyte-derived, and contribute greatly to inflammatory responses. In most situations, these two types of macrophages have different transcriptomes. However, the boundary between these two populations is not very firm. Some studies have shown that macrophages derived from monocytes can compensate for the loss of the other type of macrophages, presenting a very similar transcriptome to resident cells with little change in the original markers (47, 48). According to Zaman et al., macrophages in mouse heart can be divided into three types based on their markers and functions (49): (1) T-cell immunoglobulin and mucin domain-containing protein 4 (TIMD4) + CC-chemokine receptor 2 (CCR2) -macrophages that have the ability to self-renew, (2) major histocompatibility complex II (MHC II)^hi^ CCR2- macrophages, 30% of which are replaced by monocytes, and (3) MHC II ^hi^ CCR2+ macrophages, which are the monocyte-derived cells mentioned above. Dick et al. used fate mapping and transcriptomes to distinguish four populations classified on the basis of markers like TIMD4, lymphatic vessel hyaluronan receptor-1 (LYVE1) (48, 50–53), MHC-II, and CCR2. These macrophage subtypes are replaced in different proportions by monocytes in the adult myocardium (48). Although the M1/M2 and other classifications discussed above seem different, where the former emphasizes function and the latter the origin, some similarities and overlap can be found between them. M1 and monocyte-derived macrophages tend to function as tissue destroyers, while M2 and resident macrophages promote the reparative process and maintain stability. Similarly, macrophages in humans can also be divided into two subsets according to CCR2, CCR2–, and CCR2+ macrophages, which have analogical functions of those in mice (54).

### Distribution of Macrophages in the Heart Tissue

Immune cells are distributed throughout the human body. It has been reported that nearly all types of white blood cells are present among cardiomyocytes and in other soft tissues in adult mouse hearts (55). Among these, macrophages have been shown to be plentiful, widespread, and carry out a variety of functions (35, 54). To distinguish the different roles of macrophages, we consider them in terms of the following tissue function: electrical conduction, blood flow, and pumping.

In the cardiac electrical conduction system, the atrioventricular node connects atrial with ventricular electrical signals to maintain atrioventricular synchronization. Hulmans et al. found that at the distal atrioventricular node, macrophages are electrically connected to cardiomyocytes *via* the gap junction protein connexin 43. Ablation of connexin 43 in macrophages or elimination of macrophages disrupts the cardiac rhythm and leads to atrioventricular block (56).

Macrophages also play a crucial role in the formation and maturation of coronary arteries. Embryo-derived macrophages, located close to coronary arteries, facilitate maturation of the coronary artery system and increase blood perfusion and coronary plexus remodeling (57). Gula et al. found that during embryonic development in mice, the first cardiac tissue macrophages are located in the subepicardial area at the E10 stage, where the formation of blood and lymphatic vessels occurs (58). In 1-day-old neonatal mouse hearts, embryo-derived macrophages also play a vital role in the promotion of cardiac recovery and angiogenesis after myocardial infarction (59, 60).

With regard to structures associated with the pumping of blood, there are steady numbers of macrophages among cardiomyocytes and valves under normal conditions (55, 61). During myocardial infarction, the quantity of macrophages rapidly increases in remote zones (62, 63), which may be due to an altered microenvironment in the presence of high ventricular stress (63).

In addition to the above locations, macrophages exist in the serous fluid in the middle of the pericardium where they have essential functions that include defense against the accumulation of pathogens and leukocytes (64–66). After myocardial infarction, GATA binding protein 6 (GATA6) + macrophages in the pericardial cavity are recruited to the remote infarct area to function in a cardioprotective role (67).

### Macrophages and Heart Homeostasis

Cardiac macrophages contribute to heart homeostasis by supporting microenvironmental balance and mechanical contraction. In addition, they function in defense against pathogens, clearance of aging and dead cell debris, regulation of the extracellular matrix (ECM), and response to tissue stress (55). In Swirski and Nahrendorf's studies, they proposed that macrophages might interact with the nervous system since they could influence signals passing through synaptic junctions (55). In the cardiac electrical conduction system, macrophages can also influence the membrane potential of cardiomyocytes and conduction through connexin 43 (also known as gap junction protein alpha 1, GJA1) (68).

### Role of Macrophages in Heart Regeneration and Repair

#### Macrophages Are Crucial in Tissue Regeneration and Repair

Numerous factors can influence tissue regeneration, including oxygen stress, immune response, sympathetic and parasympathetic signaling, ECM, metabolism, and various micro RNAs (miRNAs) (69–72). In previous studies, it has been shown that regeneration of salamander limbs is blocked when macrophages are deleted, and amputated limbs do not regenerate if the severed end is covered by fibrotic tissue. When the loss of macrophages was reversed by restoring macrophages, salamanders regained the ability to regenerate body parts (73). In addition to limb regeneration, Godwin et al. also reported an indispensable role for macrophages in salamander heart regeneration (74), consistent with the results of Aurora's studies (59). These studies have shown that macrophages activate fibroblasts and regulate lysyl oxidase activity soon after heart ischemic or cryogenic injury (59, 74). In later studies using gene editing, such as Tribbles homolog 1, or drug delivery, such as clodronate liposomes, it was shown that macrophage deficiency is lethal to mammals and increases the risk of cardiac rupture (75–78).

#### Monocytes Coordinate With Macrophages During Repair

Monocytes derived from myeloid progenitors are also an important component of the immune system owing to their potential to differentiate into dendritic cells and macrophages. They can be recruited from bone marrow and extramedullary hematopoietic sites, migrating to specific destinations in response to chemokine binding to CCR2 (79). In general, monocytes in the mouse can be divided into two subsets, classical [expressing high lymphocyte antigen 6 complex (Ly6C), positive for CCR2 and low in chemokine C-X3-C motif receptor 1 (CX3CR1)] and non-classical (expressing low Ly6C, negative for CCR2 and high in CX3CR1) (36). The corresponding subsets in humans are a cluster of differentiation (CD) 14^++^CD16^−^ and CD14^+^CD16^+^ monocytes (79, 80). After an ischemic injury, abundant circulating Ly6C^high^ (CD14^++^CD16^−^) monocytes are rapidly recruited to the infarcted area, where they contribute to the initiation of neutrophil infiltration and differentiate into macrophages that remove cell debris and degrade the extracellular matrix (79, 81). In the later stage of the response, Ly6C^low^ (CD14^+^CD16^+^) monocytes, which can be derived from Ly6C^high^ monocytes (81), tend to accumulate in the area and express high levels of growth factors like vascular endothelial growth factor (VEGF) (36), contributing to tissue healing (81). Nuclear receptor subfamily four group A member 1 (Nr4a1) is crucially important for the development of Ly6C^low^ monocytes and regulation of monocyte behavior and cytokine expression by Ly6C^high^ monocytes (81, 82). In a clinical study on the association between different monocyte subsets and prognosis of patients with acute myocardial infarction (AMI), it was found that CD14^++^CD16^−^ monocytes were negatively related to the myocardial repair and recovery of left ventricular function (83).

#### Macrophages Function in Heart Regeneration and Repair

In general, macrophages are crucial for repair after injury. During tissue repair, macrophages can aggravate an ischemic injury by disturbing the microenvironment and metabolism and causing cell death. On the other hand, cytokines like vascular endothelial growth factor-α (VEGF-α), insulin-like growth factor-1 (IGF-1), produced by macrophages promote the proliferation of various cells (84).

**Figure 1:**
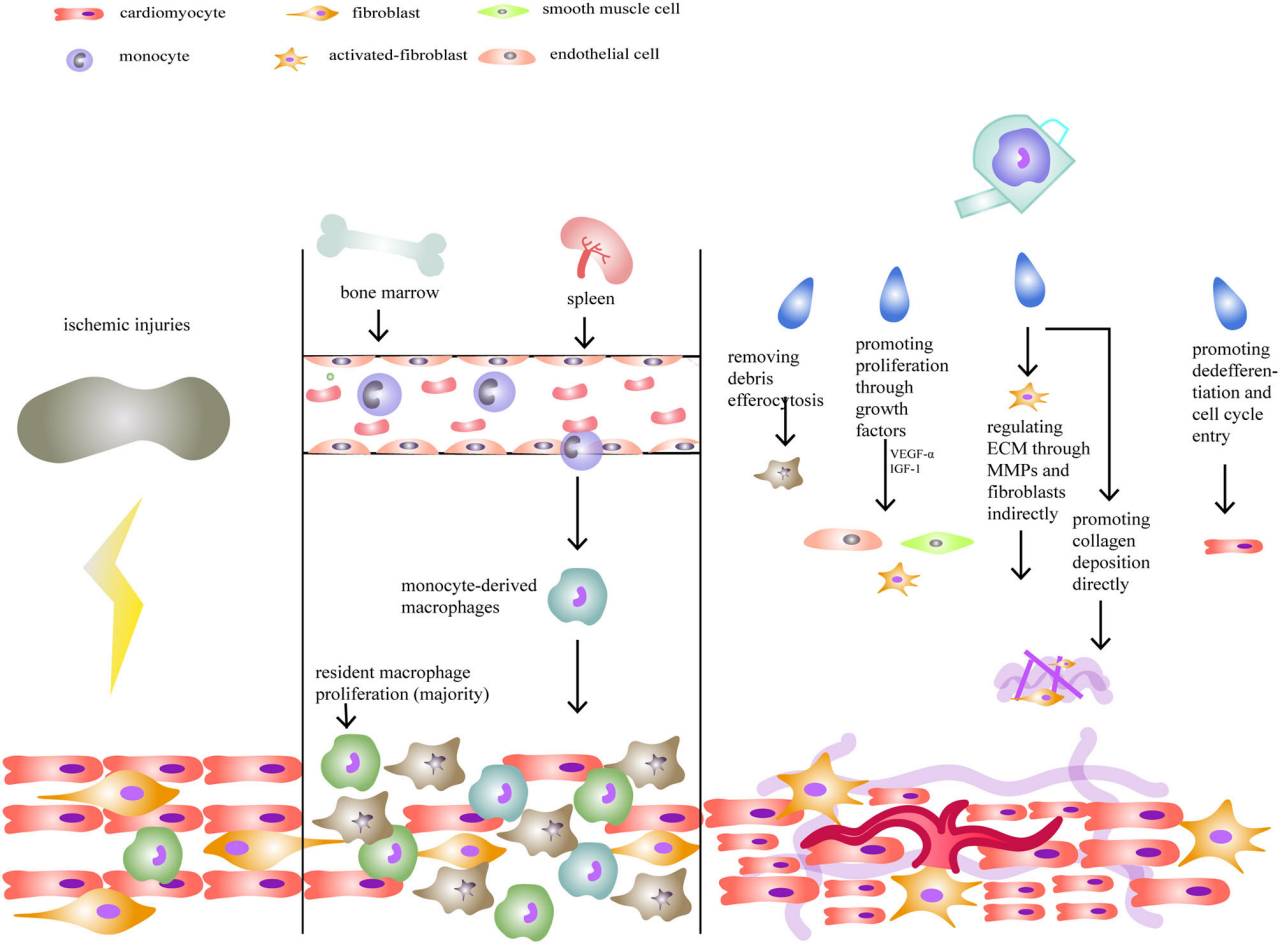
Macrophages and neonatal heart regeneration. After injuries, resident macrophages play a more important role in heart regeneration compared to monocyte-derived macrophages. Their functions include removing debris, promoting cellular proliferation, regulating extracellular matrix (ECM), and stimulating cardiomyocytes to enter the cell cycle.

In terms of heart regeneration and repair, macrophage functions can be divided into three primary categories (Figure 1). First, macrophages can stimulate the proliferation of endothelial cells, smooth muscle cells, and fibroblasts by releasing growth factors (85, 86) and promoting angiogenesis for tissue restoration. In the study of Fantin et al., cardiac tissue macrophages were shown to participate in the formation of capillary networks in the myocardium, which might be mediated by the neuropilin1 signaling pathway. Following this, macrophages can work as chaperones in tip cell fusion targeted by vascular endothelial growth factor (VEGF) (85). Moreover, it has been reported that macrophages can differentiate into endothelial cells or endothelial precursors (87, 88).

Second, ECM composition is regulated by macrophages. In an axolotl cryogenic cardiac model, researchers found that fibrosis was disrupted when macrophages were depleted, showing that macrophages are crucial for fibrosis and maturation of the ECM (74). On the one hand, by secreting TGF-β and fibroblast growth factors, macrophages indirectly activate myofibroblasts to produce collagen and work together with them to release matrix metalloproteases (MMPs) that modify ECM composition (84, 89). On the other hand, many studies have discovered that macrophages can directly promote fibrosis. In 1999, Weitkamp et al. first found macrophages, isolated from human samples, could secrete Type VIII collagen *in vitro* (90). Later, other studies also identified that macrophages could synthesize proteoglycans and related proteins (91), Type VI collagen (92, 93), which were important components of ECM. In a research published in 2020, by analyzing the transcriptome from zebrafish hearts following injury (1 day, 5 days, and 14 days) and comparing P1 and P7 neonatal mouse hearts 7 days after myocardial infarction, collagen and ECM-associated genes were found in macrophages, indicating their autonomous and direct role in fibrosis (94). In mice, macrophages produced about 6.8% of total collagen (94). Since macrophages could exhibit a fibrosis-associated phenotype, some researchers proposed that macrophages could go through a fibroblast-like transition after ischemic injury (93, 95, 96).

Third, macrophages can stimulate cardiomyocytes to dedifferentiate and enter the cell cycle (25). In neonatal humans and mice, it was revealed that resident macrophages play an important role in augmenting cardiac proliferation under low oxygen conditions (97). In salamanders, however, when macrophages were depleted in a heart cryo-injury model, whole heart regeneration was blocked when the activation of fibroblasts and remodeling of the ECM was disturbed. In contrast, cardiomyocyte proliferation was unaffected (74). Given these disparate results, the ability of macrophages to directly or indirectly promote cardiomyocyte proliferation in mice and humans requires further study.

#### Different Macrophage Subsets Have Distinct Roles in Heart Repair

When the heart is injured, different types of macrophages usually respond in a variety of ways over different time scales (98). In the early response to ischemic damage signals in mouse, numerous Ly6C^high^ monocytes are recruited from blood and lead to further M1-type macrophage activation, which provides the injured area with a “spring-cleaning” (36, 99, 100). In addition, M1-type macrophages can significantly upregulate MMP1, 3, 7, 10, 14, and 25 to modulate the ECM (41, 81, 101). Meanwhile, Ly6C^high^ monocytes and other immune cells, mainly neutrophils, secrete a series of chemokines and cytokines like IL-1β, IL-6, tumor necrosis factor (TNF)-α, and chemokine (C-C motif) ligand (CCL)-3, and CCL-4 to amplify inflammation and activate cardiomyocytes and endothelial cells (102). At later times during tissue repair, changes in the microenvironment stimulate macrophages to polarize toward the M2 type and to increase the concentration of VEGF, and myeloid-derived growth factor (MYDGF) (75, 103), which increases cell proliferation and production of new vessels (23, 59, 78). In some organs, M2 macrophages seem to be more essential for repair than the M1 type (75), and in the heart, a reduced M1/M2 ratio is associated with increased numbers of vessels and more stable ECM. However, contrary to earlier results, a recent study reported simultaneous pro-inflammatory and anti-inflammatory effects in response to cardiac cryoinjury, but not a transition from M1 to M2 macrophages (104). Also, crosstalk between M1 and M2 macrophages should not be neglected as M1 polarization may be essential for the activation of M2 (46, 105). Since lipopolysaccharide (LPS) and IL-4 can elicit different types of macrophages *in vitro*, Munoz-Rojas et al. co-stimulated macrophages with LPS+IFN-γ and IL-4, and by single-cell sequencing, they found that this kind of macrophage contained a new transcriptome. Interestingly, some cytokines like IL-6, IL12b, arginase-1 (Arg1), and chitinase-3-like protein 1 (Chi3l1) began to be exclusively expressed in macrophages (106).

As we discussed earlier, macrophages can be classified as resident or monocyte-derived. These two kinds of macrophages have distinct functions as well (47, 48, 107). Normally, both resident and monocyte-derived macrophages exist in neonatal and adult hearts, but their subtypes and quantities differ. Neonatal hearts, which can renew themselves, will selectively amplify the resident population with little increase in the numbers of monocyte-derived cells (Figure 1). In contrast, adult hearts contain increased numbers of monocyte-derived macrophages, although the percentage of resident cells is initially similar to neonates (30, 60, 63). In a cardiomyocyte ablation experiment, it was found that resident macrophages could produce less inflammation and promote cell proliferation and the formation of new coronary vessels, while monocyte-derived macrophages only had inflammatory effects (60). Also, it was shown that macrophage amplification following injury in neonatal mice occurred rapidly, over no more than 5 days, while inflammation and repair tended to be slower in adult hearts (60, 97). However, since monocyte-derived macrophages can replace the loss of resident cells, the differences between responses to injuries in neonatal and adult hearts require further study to elucidate mechanisms.

#### Different Roles of Macrophages in Ischemic and Non-ischemic Heart Disease

In ischemic heart disease, different responses are observed in infarct, peri-infarct, and remote regions (49). In the infarct zone, the number of resident macrophages is greatly reduced and numerous monocytes are recruited for the clearance of cell debris. In the peri-infarct zone, the rapid proliferation of resident macrophages contributes to myocardial repair, the loss of which can lead to negative remodeling. Finally, in the remote zone, little proliferation or change in the numbers of resident macrophages is observed. After an injury, CCR2– and CCR2+ resident macrophages contribute to the recruitment of monocytes (47). CCR2+ macrophages promote monocyte and neutrophil recruitment through damage-associated molecular patterns (DAMPS)-toll-like receptor (TLR)-myeloid differentiation factor 88 (MyD88) signaling, but in contrast, CCR2– macrophages may reduce the aggregation of monocytes (47, 108).

Macrophages play various roles in non-ischemic heart diseases, such as pressure overload hypertrophy and chronic heart failure. In a hypertension model where macrophages were depleted by clodronate liposomes, loss of heart function and fibrosis were clearly attenuated (109). When mechanical strain is exerted on the myocardium, the mitogen-activated protein kinase (MAPK) pathway can be activated, promoting re-entry to the cell cycle and increasing the proliferation of macrophages (63). Pressure-overload hypertrophy is often accompanied by the activation of sympathetic nerves and angiotensin-family proteins. It has been shown that the CXCL1-CXCR2 axis participates in the regulation of monocyte recruitment during hypertrophy (110). In the early stages of hypertrophy, resident macrophages proliferate under the influence of the transcription factor krüppel-like factor 4 (KLF4), leading to an increase in the formation of new vessels (49, 111). Monocytes then take over the role of the resident macrophages to function in cardiac protection (49).

#### New Targets for Enlisting Macrophages for Cardiac Regeneration and Repair

Since macrophages play a crucial role in heart regeneration, various factors affect the process. Cells like neutrophils (112), Foxp3+CD4+ regulatory T cells (113), cytokines like IL-4 (75), IL-35 (114), IL-10 (33, 105, 115, 116), other proteins like Dectin-2 (98), matrix metalloproteinase-28 (117), and serum-glucocorticoid regulated kinase 1 (118) can all affect the activation of macrophages or the M1/M2 ratio, and thus likely influence heart regeneration. Nuclear receptor subfamily 4 group A member 1 (NR4A1) inhibits the expression of IL-6 and TNF to promote phenotype transitions of macrophages. In recent years, great progress has been made in revealing metabolic transformations in macrophages. It has been reported that changes in energy sources, such as glucose or lipids, can affect the utilization of substrates by macrophages, as can macrophage polarization (46, 119, 120).

Given the crucial role of macrophages in cell proliferation and heart regeneration, many studies seek new targets for stimulating macrophages to more efficiently exert positive effects. Some studies have reported that anti-TNF therapy had a certain effect on cardiac function in tumor patients with heart failure (121), thus demonstrating an effect of specific anti-immune therapy. Clinically, many drugs, such as trimetazidine or sodium-glucose cotransporter-2 (SGLT2) inhibitors and anti-inflammatory therapies, such as IL-1β inhibition are proven to have an effect on improving cardiac function (46, 122–124). However, whether they may target macrophages is uncertain. In recent years, many studies have tried to investigate the potential therapy methods, such as simple compounds, nanoparticles, exosomes from macrophages (engineered and unprocessed), and mesenchymal stem cells (MSCs), targeting macrophages during cardiac injury (both ischemic and non-ischemic injuries) in animal models (77, 125–130). Compared to simple compounds, nanoparticles and engineered exosomes provide for more precise targeting, thus decreasing the side effects and enhancing the effectiveness (25). In a recent study, Rangasami et al. used nanoparticles derived from hyaluronic acid (HA) to deliver anti-cancer drugs, strengthening the effectiveness while avoiding side effects (130). This offers a new therapeutic tool as the contents of the particles can be changed to make full use of the HA shell. However, considering the fact that many markers and characteristics of macrophages are known from experiments *in vitro*, the current classification of macrophages may not allow for precisely targeting specific subsets that exist *in vivo*. In addition, the tissue environment is not the same for mice and humans. Therefore, more basic research and clinical studies are needed to explore therapeutic strategies.

## Discussion

Macrophages play an indispensable role in maintaining homeostasis and repair of injuries. It has been reported that macrophages constitute the majority of leukocytes that accumulate in the heart after myocardial infarction (63). Therefore, probing the functions of macrophages and their molecular mechanisms is necessary for the study of heart injuries. Historically, there has been disagreement among researchers about the origins, subtypes, and functions of macrophages. Now, through more advanced techniques, a much clearer understanding is emerging. Compared with M1 and monocyte-derived macrophages, M2 and resident macrophages seem to have more positive effects on heart regeneration and repair. Among their many functions, macrophages also support arterial elasticity and tension (52) and promote angiogenesis and myofibroblast activation. However, from our perspective, crosstalk between different types of macrophages should not be neglected in the context of heart repair. In the future, we look forward to more therapeutic strategies being developed to target macrophages directly or indirectly, strengthen heart regeneration in neonatal mammals, and improve cardiac repair in adult hearts following injury.

## Author Contributions

YG: conceptualization and writing the original draft. NQ: data collection and paper correction. JX: data collection and paper correction. YW: writing and review and editing. All authors contributed to the article and approved the submitted version.

## Funding

This study was supported by the National Natural Science Foundation of China (No. 81670235 to YW).

## Conflict of Interest

The authors declare that the research was conducted in the absence of any commercial or financial relationships that could be construed as a potential conflict of interest.

## Publisher's Note

All claims expressed in this article are solely those of the authors and do not necessarily represent those of their affiliated organizations, or those of the publisher, the editors and the reviewers. Any product that may be evaluated in this article, or claim that may be made by its manufacturer, is not guaranteed or endorsed by the publisher.
